# A Review of Current Developments in Functionalized Mesoporous Silica Nanoparticles: From Synthesis to Biosensing Applications

**DOI:** 10.3390/bios14120575

**Published:** 2024-11-27

**Authors:** Jiaojiao Zhou, Chen Liu, Yujun Zhong, Zhihui Luo, Long Wu

**Affiliations:** 1 National R&D Center for Se-Rich Agricultural Products Processing, School of Modern Industry for Selenium Science and Engineering, Wuhan Polytechnic University, Wuhan 430023, China; jiaojiaozhou@whpu.edu.cn; 2 School of Food Science and Engineering, Wuhan Polytechnic University, Wuhan 430023, China; chen.liu@whpu.edu.cn; 3 Guangxi Key Lab of Agricultural Resources Chemistry and Biotechnology, College of Chemistry and Food Science, Yulin Normal University, Yulin 537000, China; 18778837076@163.com; 4 School of Food Science and Engineering, Key Laboratory of Tropical Fruit and Vegetables Quality and Safety for State Market Regulation, Hainan University, Haikou 570228, China

**Keywords:** mesoporous silica nanoparticles, biosensing, synthesis methods

## Abstract

Functionalized mesoporous silica nanoparticles (MSNs) have been widely investigated in the fields of nanotechnology and material science, owing to their high surface area, diverse structure, controllable cavity, high biocompatibility, and ease of surface modification. In the past few years, great efforts have been devoted to preparing functionalized MSNs for biosensing applications with satisfactory performance. The functional structure and composition in the synthesis of MSNs play important roles in high biosensing performance. With the development of material science, diverse functional units have been rationally incorporated into mesoporous structures, which endow MSNs with design flexibility and multifunctionality. Here, an overview of the recent developments of MSNs as nanocarriers is provided, including the methodologies for the preparation of MSNs and the nanostructures and physicochemical properties of MSNs, as well as the latest trends of MSNs and their use in biosensing. Finally, the prospects and challenges of MSNs are presented.

## 1. Introduction

The application of nanobiotechnology to the field of biosensing has greatly promoted the advancement of various nanosystems. One of the key aims in the development of nanoscale biomaterials is to explore high-performance advanced nanomaterials. As an emerging class of nanomaterials, mesoporous silica nanoparticles (MSNs) exhibit bioactive behavior [1] due to their silanol groups [2], enabling their use in the manufacture of nanocomposites for biomedical applications.

The properties of nanomaterials are related to their structures, and the application of MSNs has been fueled by the following features: (1) their particle diameter can be regulated according to the preparation conditions, ranging from several nanometers to a few micrometers. Monodisperse nanoparticles can be obtained in a size range from 10 to 300 nm, which is relevant for biological environments, allowing the optimization of the particle size for specific applications [3]. (2) The pore size is a key parameter for the molecules that can be introduced into the mesopores. According to the surfactant and synthesis conditions, the pore size can be adjusted to between 2 and 30 nm. Based on this property, MSNs with a large pore diameter can be used for the adsorption and delivery of biomolecules [4]. (3) The pore volume of traditional MSNs is calculated to be within a range from 1 cm^3^/g to 4.5 cm^3^/g [5]. MSNs are well known for their capacity to host a great number of molecules [6]. (4) MSNs were shown to possess different porous morphologies and textures, such as cubic and radial porosity, and controlling the morphology of MSN pores has been reported, allowing the selective loading of different molecular cargoes or tuning the cargo’s release [7]. (5) MSNs can be prepared and mass-produced from commercial precursors. So far, various types of MSNs have been proven to effectively co-deliver therapeutic agents, including core-shell MSNs and hollow MSNs (HMSNs) [8]. The excellent performance of MSNs in biosensing applications has promoted the development of new advanced multifunctional materials. In this sense, the continuous progress of nanotechnology has promoted the development of nanoparticles that can establish close interactions in the biological world. As mentioned above, the chemical properties of the surfaces of MSNs can easily be adjusted. Many surface modification strategies have been reported [9]; host–guest interactions can now be designed to achieve multifunctional nanocarriers.

**Scheme 1:**
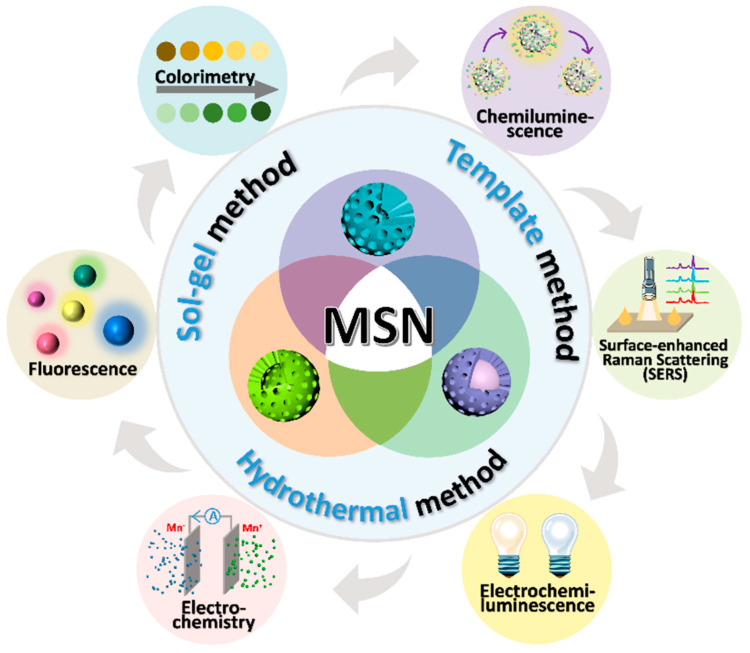
The synthesis methods of functionalized mesoporous silica nanoparticles for biosensing applications. MSN: mesoporous silica nanoparticles.

MSNs have been widely used to fabricate biosensors due to their unique properties (Scheme 1). A timely review article on this topic is necessary because a large amount of research on MSN-based sensors has now been published. In addition, great success has been achieved in preparing MSN-based materials with different nanostructures, it is necessary to review the various techniques developed for synthesizing MSNs. Herein, we focus on the latest progress in MSN preparation and their applications in biosensing. The challenges and the future prospects of MSNs are also discussed.

## 2. Synthesis of Mesoporous Silica

Various methods can be used to synthesize MSNs with multiple shapes and physicochemical properties, but all of them are based on the hydrolysis and condensation of silica precursors [9]. MSNs are often used as drug carriers to achieve the rapid release, slow release, and controlled release of drugs, due to their unique mesoporous pore size and adjustable nanopore structure [10]. However, when used for drug delivery, traditional silica nanoparticles cannot be easily metabolized in the human body, thus greatly limiting drug loading due to their pore size [11]. To solve this problem, researchers have developed methods for fabricating dendritic mesoporous silica nanocomposites (DMSNs) [12,13] and hollow mesoporous silica nanoparticles (HMSNs) [14,15] by controlling some basic parameters. By using these methods, the size of silicon nanoparticles can be controlled from within a few micrometers to several nanometers, enabling significant changes in their physicochemical, surface, and structural properties, thereby greatly expanding the application field of silicon nanomaterials.

Mesoporous materials with inorganic substances as skeletons are the products of coordination between self-assembly and the sol-gel reaction. In the self-assembly process, the inorganic framework materials are assembled on the surface of the template micelles, while in the sol-gel process, the assembled framework materials are fixed in situ to generate mesoporous products. Different methods, including the sol-gel method, hydrothermal method, and template method, have been proposed to fabricate MSNs.

### 2.1. Sol-Gel Method

**Figure 1:**
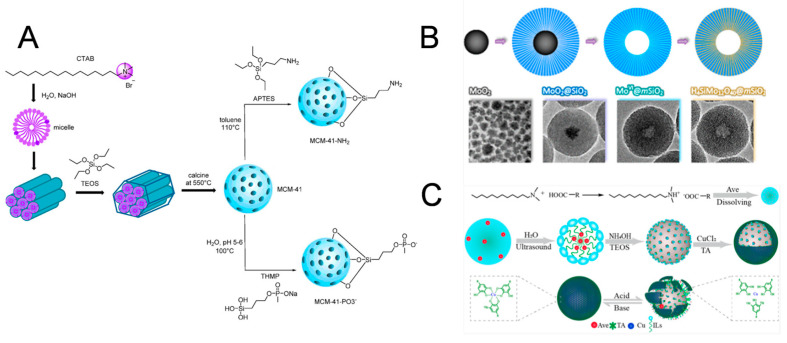
Various synthesis strategies of MSNs. (**A**) Synthesis of functionalized MCM^−^41 via a sol-gel process, adapted from Ref. [21] with the permission of the American Chemical Society. (**B**) Scheme of an inside-out preinstallation-infusion-hydration method for the targeted synthesis of Keggin heteropoly acids within MSNs, adapted from Ref. [25] with the permission of the American Chemical Society. (**C**) Scheme of preparation and the possible release mechanism of Ave-IL@MSN@TA-Cu microcapsules, adapted from Ref. [26] with the permission of the American Chemical Society.

The sol-gel method involves the addition of active precursors to a solvent, which then polymerizes to a gel at a specific temperature to produce mesoporous nanomaterials with a uniform pore size [16]. This method reacts easily under homogeneous mixing and the loaders are diffuse in the nm range. However, this method presents some disadvantages because it is time-consuming and harmful to health. Moreover, the resulting gel would be subject to shrinkage during drying. The synthesis of monodisperse nanoparticles was first developed by Stöber and involves the hydrolysis of tetra alkyl silicate in a mixture of ethanol and water under the catalysis of ammonia [17]. The preparation of MSNs mainly follows the Stöber method, which is also known as the sol-gel method. Specifically, the sol phase is generated by the reaction of hydrolysis and condensation, with high surface activity compounds as precursors at alkaline or acidic pH, while the gel phase is a three-dimensional structure produced by the condensation of colloidal particles through the cross-linking of siloxane bonds [18,19]. Using surfactants, many types of MSNs can be designed by changing the reactants. As early as 2012, MSNs with a uniform pore size were synthesized for the first time by Shi’s team [20], using cetyltrimethylammonium chloride (CTAC) as the structure-directing agent, tetraethyl orthosilicate (TEOS) as the silica precursor, and triethanolamine (TEA) as the alkaline catalyst, achieving the size adjustment of MSNs by changing the amount of TEA being added. In another study by Ang et al. [21], MSNs were synthesized as drug carriers using a sol-gel method, and their surface was further modified with amine and phosphonate groups. Specifically, MSNs were achieved using surfactant cetyltrimethylammonium bromide (CTAB) as the structure-directing agent, TEOS as the silica source, and NaOH as the base catalyst. The preparation of both pristine and functionalized MSNs is shown in Figure 1A. In addition, researchers have investigated whether changes in basic parameters, such as temperature and pH value, can adjust the size of MSNs. In a study by Tella et al. [22], the effects of crux reaction conditions (such as the amount of TEOS, pH value, and reaction time) on the synthesized MSNs were systematically considered, and the pH value was concluded to be the most important factor affecting particle size. The sol-gel method is the most frequently used technique to prepare MSNs, offering the advantages of producing a uniform structure, controllable performance, and saving time (due to the use of fewer excipients).

### 2.2. Hydrothermal Method

In the hydrothermal technique, synthesis is typically performed by the chemical reaction of substances in a sealed container and heated aqueous solution at a high temperature and pressure [23]. This approach has several advantages, including ease of controlling the reaction and the formation of metastable substances for an adequate reaction. However, this method has the disadvantages of harsh demands on the equipment and unmonitored real-time processes. Specifically, a surfactant is used as a template with an acid or base catalyst, which is then added to the solution to generate a hydrogel, followed by placing the hydrogel in an autoclave. The treatment of the reaction precursors with high temperature and pressure can be described as a process of separation of the precursors and the removal of organic substances [24]. In a study by Dou et al. [25], an inside-out preinstallation-infusion-hydration method for the targeted synthesis of Keggin heteropoly acids within MSNs was reported. Discrete molybdenum dioxide (MoO_2_) nanoparticles were first prepared by a one-pot hydrothermal route; these were then used as cores to grow shells of supramolecular templated silica with TEOS and CTAC in an alkaline solution. With the thermal treatment of the as-synthesized core-shell spheres, the organic template was burned off and a mesoporous shell was formed (Figure 1B). The hydrothermal method is similar to the sol-gel procedure, except that right after template removal, the mixture is transferred to an autoclave lined with polytetrafluoroethylene at a definite temperature. The MSNs prepared by this method exhibit better performance in terms of regularity and hydrothermal stability. The hydrothermal method has been extensively employed to fabricate silica-related nanomaterials, especially for the synthesis and after-treatment of mesoporous silica to ameliorate the regularity of its mesoporous pattern and amplify the pore size.

### 2.3. Template Method

The template synthesis method, also known as the surfactant-assisted synthesis technique, is used to fabricate porous nanomaterials. This method can accurately control their size, shape, structure, and properties. However, it requires complex template removal and is commonly regarded as less efficient [12]. Up to now, various types of synthesis strategies based on soft, hard, and self-template utilization and removal have been developed. In these techniques, templates are used as structure-directing agents to produce hollow porous structures.

In the soft template method, silica nanomaterials are prepared using single micelles, micro lotion, vesicles, and bubbles as templates, followed by removing the templates by simple centrifugal washing. It is a relatively simple method for the fabrication of hollow silica nanoparticles, but it is difficult to produce nanoparticles with satisfactory dispersion and regulate the particle size and shell thickness over a wide range. It is also laborious to prepare nanoparticles on a large scale because of the demand for abundant surfactants during the synthesis process [24]. In addition, it is challenging to eradicate the template while retaining a good dispersion of nanoparticles. The residues may also have some undesirable side effects in biomedical applications. The soft-template method is widely used for double-shelled silica microcapsules with a stimulus response. For instance, Zhou et al. prepared double-shelled silica microcapsules of pesticides using N,N-dimethyldodecan-1-aminium nonanoate as the soft-template agent and solvent to entrap avermectin (Ave) inside MSNs in situ [26]. Subsequently, a tannic acid–Cu (TA–Cu) complex was employed as a sealing agent to cover the surface of Ave-loaded silica to form Ave-IL@MSN@TA-Cu microcapsules (Figure 1C). This process did not involve the removal of templates.

In the hard template method, prefabricated rigid particles are used to induce the growth of SiO_2_ on their surface, followed by the treatment of calcination or by etching removal of the templating agent after the formation of the shell layer [22]. Different kinds of hard templates need different removal methods: Na_2_CO_3_ solution is used for the etching removal of a silicon oxide template, while HCl solution is used for the etching removal of a calcium carbonate template. The size and morphology of the hard template can be designed in advance, which endows it with good stability and can effectively control the particle morphology and cavity volume.

In recent years, researchers have developed a method for preparing HMSNs without extra templates, which is known as the self-template method. In the absence of any surface protection, selective etching of the interior of silica nanoparticles to obtain hollow structures can be achieved. Several studies have shown that solid silica nanoparticles can be transformed into hollow structures after acid and high-temperature treatment. The outermost layer of silica nanoparticles by the Stöber reaction is the hardest, due to the condensation of silicic acid and its aggregates, while the innermost layer of the porous structure with a high swelling degree is the softest, allowing the thermal selective etching of the inner layer [27].

When preparing mesoporous silica nanoparticles, the pore structure, size, operation difficulty, and experimental cost of the required nanoparticles should be considered when choosing an appropriate preparation method. The properties of nanoparticles obtained by diverse synthesis methods vary slightly, and the current optimal fabrication techniques can be selected after comparison.

## 3. Biosensing Applications of Mesoporous Silica Nanoparticles

Its unique ordered mesoporous structure, controllable pore size, and the above-mentioned properties have enabled the wide application of mesoporous silica. To date, the application of mesoporous silica has been explored in the field of biosensing. Herein, we mainly focus on the various applications of MSNs in biosensing. These biosensors can be mainly divided into six types: colorimetry, fluorescence, electrochemistry, electrochemiluminescence, surface-enhanced Raman scattering (SERS), and chemiluminescence.

### 3.1. Mesoporous Silica-Based Colorimetric Biosensors

Colorimetric biosensors have received much attention in analytical chemistry, owing to their suitability for unaided visual observation, simplicity, low cost, and having no need for any complicated apparatus, suggesting their potential for on-site detection. In various colorimetric systems, the enzymatic oxidation of chromogenic substrates has been widely used in colorimetric biosensing due to its label-free, rapid, and naked-eye observable properties. Recently, nanozymes have garnered great interest. Compared with natural enzymes, nanozymes are more stable, more controllable, and easier to prepare. MSNs are ideal support matrices for optical sensing, in which these excellent adsorbents, including nanozymes [28], indicators [29], etc., could be conveniently immobilized on the interior or exterior surface of the mesoporous framework. In addition, the inert nature of the silica matrix compared to the external environment facilitates the creation of a fascinating platform on which to construct robust, sensitive, and rapid colorimetric sensors.

In a study by Norton et al. [29], a colorimetric sensor was prepared for in situ NO_3_^−^ detection, based on a square-planar platinum(II) salt supported on MSNs. When exposed to aqueous NO_3_^−^ with a pH of ≤0, the color and luminescence of platinum salts undergo distinct changes, due to the substitution of PF_6_^−^ by NO_3_^−^. This change in the photophysics of the platinum salts is caused by their lattice rearrangement, resulting in prolonged Pt… Pt… Pt interactions, accompanied by changes in electronic structure. Furthermore, coupling this material with MSNs can enhance detection sensitivity. The colorimetric sensor has high selectivity for aqueous NO_3_^−^ with the existence of other interfering anions, indicating its potential for NO_3_^−^ detection. Similarly, MSN chemosensors have been used for the detection of other ions, such as cadmium ions [30], copper ions [31], mercury ions [32], etc. However, single sensors can only detect a single analyte, which does not meet the need for high-throughput discrimination, due to the similar structures of a series of target analytes.

Sensor arrays based on nanomaterials have become powerful tools for detecting a series of analytes with similar structures. Coupling the merits of sensor arrays with a nanozyme–TMB system, a colorimetric sensor array for recognizing monoamine neurotransmitters was created by Wu et al. [33]. In their work, DMSN embedded with three metal nanoparticles (Ag/Au/Pt) was used for the detection of six monoamine neurotransmitters. These nanocomposites acted as nanozymes to oxidize TMB. Different monoamine neurotransmitters have different inhibitory effects on the catalytic efficiency of nanoenzymes, resulting in different color changes in the nanozyme–TMB system. These color-change patterns provide the fingerprint responses of monoamine neurotransmitters, which can be distinguished through linear discriminant analysis. Using this sensor array, monoamine neurotransmitters were successfully discriminated, with detection limits ranging from 0.03 to 0.38 μM, allowing human serum spiked with different monoamine neurotransmitters to be directly differentiated by a fingerprint response. This sensor array may hold great promise in practical applications, due to its outstanding discrimination performance with biofluids.

**Figure 2:**
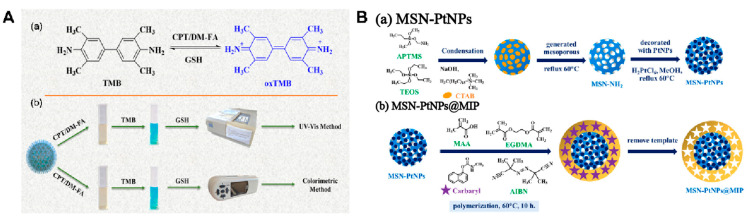
(**A**) UV–vis method and colorimetric method for GSH detection based on CPT-DM-FA, adapted from Ref. [34] with the permission of the American Chemical Society. (**B**) An MIP sensor based on MSN-PtNPs, manufactured by coating a carbaryl imprinted polymer for carbaryl assays, adapted from Ref. [35] with the permission of Elsevier.

Based on the reductive ability of thiolated compounds, colorimetric sensing could be achieved through the color change of chromogenic substrates during the redox reaction. In a study by Zhang et al. [34], an ultrasensitive core–shell nanozyme was prepared, with DMSN as a core and MnO_2_ as a shell, for glutathione (GSH) sensing and cancer detection (Figure 2A). The DMSN core not only serves as a carrier for camptothecin but also provides a matrix for loading MnO_2_ shells. Owing to the high dispersion of MnO_2_ on the interior and exterior surface of the DMSN, the atomic utilization efficiency of MnO_2_ was significantly improved, leading to increased catalytic activity, enabling the catalysis of the oxidation of TMB into oxTMB. Based on the oxidase-mimic property of MnO_2_, a colorimetric sensor was established for GSH sensing in a linear range of 5–80 μM. After surface folic acid modification, this nanocomposite could specifically detect cancer cells from 25 to 150,000 through the oxidation of TMB. This nanozyme opens up the new possibility of modulating its activity for biomedical applications.

Despite many reports of sensors based on the nanozyme–TMB strategy, there are few reports on detection strategies that combine the specificity and selectivity of MSNs with nanozyme-catalyzed visual detection. In a study by Amatatongchai et al. [35], an origami 3D-μPAD was designed for colorimetric carbaryl detection, based on MSN-PtNPs coated with a molecularly imprinted polymer (MSN-PtNPs@MIP) (Figure 2B). Coating an MIP shell on the surface of MSNs endows the composites with numerous imprinting sites for the sensitive and selective determination of carbaryl. The as-prepared MSN-PtNPs@MIP not only acted as a nanozyme for the catalysis of TMB with the existence of H_2_O_2_ but also provided cavities for selective carbaryl binding. Upon binding with carbaryl within the 3D-μPAD detection zone, a color change was observed from dark blue to light blue. This origami 3D-μPAD-based assay achieved a detection limit of 1.5 ng/g for the carbaryl assay.

The utilization of mesoporous silica-based colorimetric biosensors has drawn wide research attention, yet most of them are still in their infancy. Up to now, the most widely used point-of-care detection items are the glucose meter and pregnancy test strip. The realization of point-of-care detection would be a pivotal step in colorimetry. Furthermore, the issues of food safety, disease diagnosis, and environment monitoring have prompted an urgent need for portable sensing methods. In this regard, the rational design of corresponding meters and kits founded on the stability of MSN-based biosensors would be able to tackle this challenge.

### 3.2. Mesoporous Silica-Based Fluorescent Biosensors

Due to their high surface area, MSNs are generally used as fluorescence carriers in fluorescence assays. These fluorescent materials, such as QDs and fluorescence dyes, are commonly used for loading in MSNs.

In a study by Huang et al. [36], a novel strategy was proposed using a bright QD assembly technique within MSNs, wherein mercapto-terminated MSNs could be used to load QDs via thiol-metal coordination. Dentritic-SiO_2_/QDs/SiO_2_ spheres were combined with a lateral flow strip assay for an ultrasensitive immunoassay of the C-reaction protein in biological samples. Similarly, this novel MSN-QDs system was utilized as a convenient detection platform for other analytes, such as the rabies virus [37] and ochratoxin A [38]. Specifically, these MSNs were prepared via a template method. Then, the pore channels were used to carry QDs. Abundant QDs could be incorporated into their radial pores to improve the sensitivity of MSN-based biosensing. Furthermore, the MSN-QD complex is highly stable, even after several months of storage. Recently, Yang et al. explored the potential of thiol-capped CdZnTe QDs as an exceptional signal tag for fluorescence biosensing [39]. The NAC-capped CdZnTe QDs (NAC-CdZnTe QDs) exhibited superior anti-interference capabilities and storage stability across various temperatures, pH levels, and storage durations. Similarly, Huang et al. also reported a facile one-pot strategy to prepare an MSN-QDs system in which QDs were confined into MSNs [40]. The prepared QDs@MSNs exhibited excellent fluorescence intensity, water solubility, and stability.

**Figure 3:**
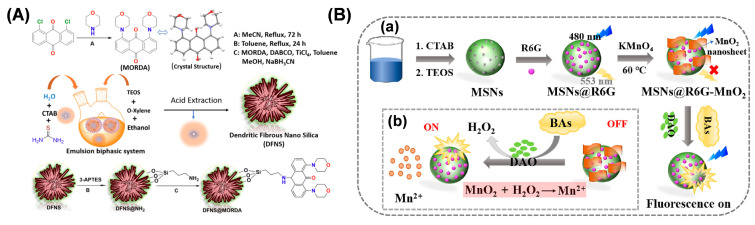
(**A**) Scheme for the synthesis of the ligand, MSNs, and the final material, adapted from Ref. [41] with the permission of Elsevier. (**B**) Scheme of a fluorescent biosensor for the determination of biogenic amines based on MSNs@R6G-MnO_2_, adapted from Ref. [42] with the permission of Elsevier. The images of (a) and (b) represent the preparation of MSNs@R6G-MnO_2_ and the reaction between Mn^2+^ and H_2_O_2_, respectively.

To increase MSN surface area for the immobilization of multifunctional payloads, uniformly sized and high-surface-area MSNs were prepared, using thiourea as the hydrolyzing agent, by Yadav et al. [41]. Subsequently, MSNs and 1,8-dimorpholinoanthracene-9–10-dione were used to detect and remedy Cu^2+^ (Figure 3A). Quenching behavior was observed, based on the fluorescence resonance energy transfer (FRET) in the presence of Cu^2+^, achieving a detection limit of 0.13 µM. The produced material could adsorb Cu^2+^ with a maximum adsorption capacity of 398 mg/g, coupled with easy recycling.

Based on rhodamine 6G-loaded (R6G) and MnO_2_ nanosheet-coated MSNs, a fluorescence probe was designed for the detection of biogenic amines by Zhang et al. [42]. In the presence of biogenic amines, diamine oxidase will catalyze them to generate H_2_O_2_, leading to the reduction of MnO_2_ to Mn^2+^ and the signal recovery of R6G (Figure 3B). A good recovery rate was obtained with spiked fish samples using the analytical method, revealing its potential application in food safety.

### 3.3. Mesoporous Silica-Based Electrochemical Biosensors

Electrochemical sensors have garnered much interest, owing to their low cost, high sensitivity, fast response, and ease of miniaturization [43,44,45,46]. Most importantly, electrochemical sensors have the unique merit of offering both labeled and unlabeled detection [47,48].

**Figure 4:**
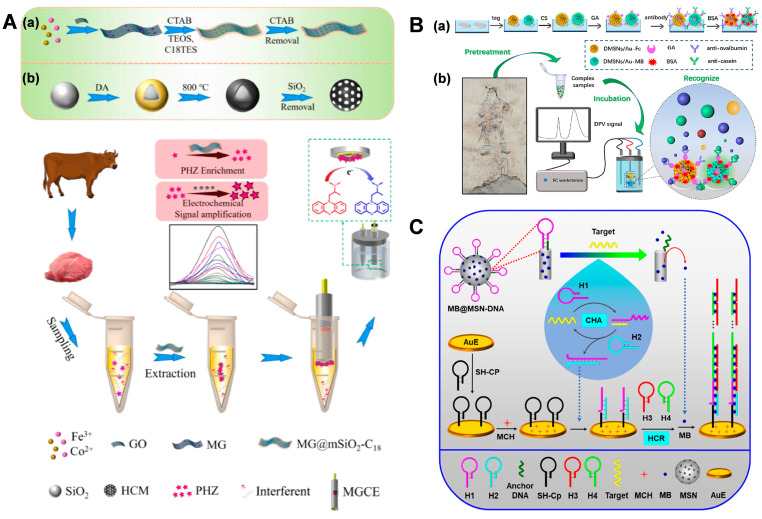
(**A**) Scheme of the electrochemical detection of PHZ, adapted from Ref [49] with the permission of Elsevier. The images of (a) and (b) represent the preparation of CoFe_2_O_4_/graphene coated with C_18_-functionalized mesoporous silica and the vertical coating of organic-inorganic hybrid mesoporous silica on nitrogen-doped hollow carbon microspheres (HCM), respectively. (**B**) Scheme of the multiplexed mechanism on the electrochemical immunosensor, adapted from Ref [61] with the permission of Elsevier. The images of (a) and (b) represent the preparation of signal nanotags based on different Au modified dendritic MSN and the electrochemical detection of eggs and milk proteinaceous binders used in ancient wall paintings, respectively. (**C**) Scheme of the electrochemical biosensor for miRNA detection, adapted from Ref [63] with the permission of the American Chemical Society.

To avoid interference during detection, Yang et al. integrated an electrochemical sensor with magnetic solid-phase extraction for promethazine (PHZ) measurement in meat samples [49]. Firstly, CoFe_2_O_4_/graphene was coated with C18-modified mesoporous silica (MG@mSiO_2_-C18) to extract the PHZ, while a magnetic glassy carbon electrode modified with nitrogen-doped hollow carbon microspheres (HCM) was used to attract MG@mSiO_2_-C18 with the PHZ, achieving the direct detection of PHZ without an elution procedure. As a result, this method exhibits a detection limit of 9.8 nM for a PHZ assay (Figure 4A).

The large number of silanol groups or organo-functional groups in MSNs provide a means to anchor gating molecules to cap the pores, including DNA [50,51], proteins [52], and nanoparticles [53]. These gating molecules can also uncap the pores by various external stimuli, such as magnetic fields [54], pH [55], enzymes [56], and target molecules [57,58]. Based on the electrochemical properties of Ag anodic stripping and the high volume-to-surface area of MSNs, Shi et al. proposed an MSN/AuNP@Ag-based electrochemical lateral flow immunoassay for rapid AFP detection [59]. MSNs/AuNPs@Ag were prepared by loading an Ag shell into a AuNP-coated DMSN. The electrochemical signal was significantly enhanced upon anodic stripping. Moreover, the conjugation of AuNPs with the Ag shell and the antibody facilitated efficient signal amplification. This developed electrochemical biosensor has two advantages. First, the electrochemical signal allows for both qualitative and quantitative analysis. Second, this biosensor holds substantial potential for home-based diagnosis. This study not only offers a reference for the establishment of ultrasensitive bioassays but also provides a prototype of a portable sensor.

Compared to other detection methods, a simultaneous detection method has drawn attention due to its shorter analysis time [60]. On the basis of different signals being tag-loaded onto the surface of Au-modified dendritic MSNs (DMSNs/Au), Sun et al. developed an electrochemical immunosensor for the simultaneous determination of ovalbumin from egg white and casein from milk in wall paintings [61]. DMSNs not only adsorbed more electrochemical probes but also immobilized more antibodies, thus significantly improving the sensitivity of the immunosensor. As a result, the fabricated immunosensor achieved detection limits of 0.59 ng/mL and 0.36 ng/mL, respectively (Figure 4B).

To obtain a low background in electrochemical biosensing strategies, Cheng et al. proposed an MSN-based sensing platform for mRNA detection through exonuclease-induced target recycling amplification [62]. However, this nuclease-based amplification strategy might suffer from inhibiting factors in complex sample analysis.

Alternatively, a non-enzyme-based amplification strategy is highly desirable. Previous authors proposed an ultrasensitive electrochemical sensing platform with a low background for microRNA (miRNA) detection based on MSN containers, catalytic hairpin assembly (CHA), and hybridization chain reaction (HCR) amplification [63]. Initially, methylene blue (MB) would be sealed in MSNs by DNA. The presence of a target would induce the release of MB due to the base paring reaction, coupled with the simultaneous occurrence of CHA target recycling. Meanwhile, the CHA products could be captured and would then trigger the HCR process to produce double-stranded DNA, which could be employed for MB intercalation. In this way, an increased electrochemical signal could be recorded (Figure 4C). Based on the nonenzymatic amplification strategy and low background, the electrochemical platform could achieve a detection limit down to 0.037 fM.

In summary, electrochemical biosensors based on MSNs have shown high sensitivity and selectivity. The development of various approaches has expanded the application of MSNs and offers new avenues for research in this field.

### 3.4. Mesoporous Silica-Based Electrochemiluminescent Biosensors

Electrochemiluminescence (ECL), also defined as electrogenerated chemiluminescence, is a process wherein a high-energy electronic state is excited through an electrochemical process [64]. A common strategy for constructing MSN-based ECL biosensors is the use of MSNs as carriers to wrap ECL emitters for hole sealing. Coupled with biorecognition molecules such as aptamers and antibodies, these nanocomposites can recognize specific target molecules, leading to the changing of ECL emitters, followed by an ECL signal change [65,66].

**Figure 5:**
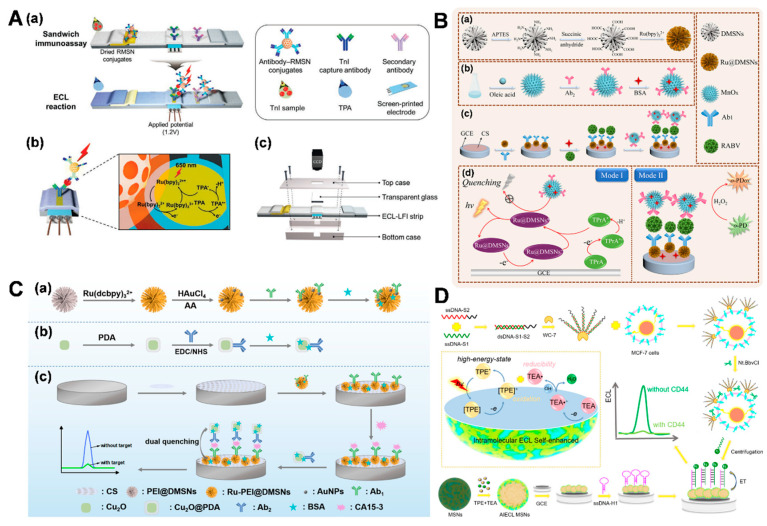
(**A**) Scheme of the structure of ECL-LFI, adapted from Ref. [67] with the permission of Wiley. (**B**) Scheme of a dual-mode immunosensor for detecting the rabies virus, adapted from Ref. [68] with the permission of the American Chemical Society. (**C**) Scheme of a dual-quenching ECL immunosensor for CA15-3 detection, based on Ru-PEI/AuNPs@DMSNs and Cu_2_O@PDA, adapted from Ref. [69] with the permission of the American Chemical Society. (**D**) Scheme of CD44 extraction and ECL sensor fabrication, adapted from Ref. [70] with the permission of the American Chemical Society.

In a study by Hong et al. [67], tris(2,2′-bipyridyl)ruthenium(II) (Ru(bpy)_3_^2+^) was loaded into MSNs and the troponin I (TnI) antibody was attached to their surface. When Ru(bpy)_3_^2+^ reacted with tripropylamine, a strong ECL signal was produced that could be monitored. This ECL lateral flow immunosensor (ECL-LFI) could successfully detect TnI-spiked human serum within 20 min at femtomolar levels (Figure 5A). Therefore, MSNs provide a new avenue for developing effective ECL-LFI biosensors for biomarker detection.

Donor-acceptor creation is an effective strategy to improve assay sensitivity. Li et al. developed an ECL resonance energy transfer (ECL-RET) immunosensor using Ru@MSNs as ECL emitters and manganese oxide nanoparticles (MnOx) as quenchers for sensitive detection of the rabies virus [68]. Ru@DMSNs were obtained by loading Ru(bpy)_3_^2+^ into DMSNs as ECL emitters. MnOx can inhibit the formation of Ru@DMSN excited states, accompanied by a decreased ECL signal. RABV quantification can be realized through the variation of the ECL signal (Figure 5B). In another work, Wang et al. constructed an ECL immunosensor for CA15-3 detection, based on a dual-quenching strategy [69]. Briefly, Ru(dcbpy)_3_^2+^, poly-(ethylenimine) (PEI), and AuNPs were immobilized on DMSNs (Ru-PEI/AuNPs@DMSNs) as luminophores. Upon the addition of CA15-3, Cu_2_O nanoparticles coated with poly(dopamine) (Cu_2_O@PDA) nanocomposites were introduced to the as-prepared Ru-PEI/AuNPs@DMSNs via an antigen–antibody interaction, causing remarkable ECL quenching due to the dual quenchers of Cu_2_O and PDA (Figure 5C). The fabricated sensor could detect CA15-3 within a wide linear range of 5.0 × 10^−5^–6.0 × 10^2^ U/mL.

Aggregation-induced ECL (AIECL) has introduced new vitality into the preparation of ECL emitters. In a study by Jia et al. [70], an AIECL biosensor was proposed using MSN matrix-confined 1,1,2,2-tetra(4-carboxylphenyl)ethylene (TPE) as an ECL emitter. TPE and its co-reactant, TEA, were encapsulated in the MSNs to create a self-enhanced ECL system. This self-designed WHPWSYC (WC-7) heptapeptide could significantly reduce steric hindrance in CD44 affinity tests. Combined with the DNA strand displacement reaction, this method exhibits a good ECL response to the CD44 antigen (Figure 5D).

### 3.5. Mesoporous Silica-Based SERS Biosensors

Surface-enhanced Raman scattering (SERS) can provide comprehensive information about molecular vibration characteristics and can reflect the actual molecular fingerprint [71,72,73]. The SERS technique has garnered much attention in various fields, from biosafety to security screening [74,75,76].

**Figure 6:**
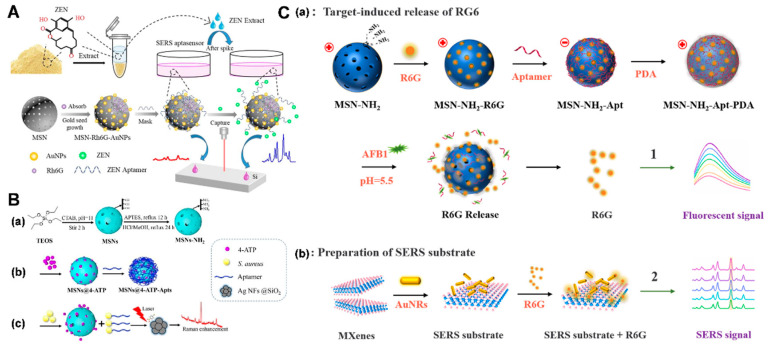
(**A**) Scheme of a SERS aptasensor for ZEN detection, adapted from Ref. [77] with the permission of Elsevier. (**B**) Scheme of *S. aureus* detection based on aptamer-gated MSNs, adapted from Ref. [78] with the permission of the American Chemical Society. (**C**) Scheme of the dual signal-on biosensor for SERS and the fluorescence detection of AFB1, adapted from Ref. [81] with the permission of Elsevier.

Guo et al. prepared MSN-loaded gold nanocomposites and Rhodamine 6G (R6G) as a SERS substrate (MSN-Rh6GAuNPs) for zearalenone (ZEN) detection [77]. The small nanogaps between the AuNPs enabled the MSN-Rh6G-AuNPs to exhibit a strong SERS signal, while the aptamer was used for ZEN recognition and Raman signal masking (Figure 6A). Similarly, Zhu et al. designed a SERS biosensor for *Staphylococcus aureus* (*S. aureus*) determination, based on MSNs [78]. Positively charged MSNs were used to immobilize the signal molecules. Then, the aptamers of *S. aureus* were assembled on the surface of MSNs via electrostatic interactions. In the presence of *S. aureus*, the assembled aptamers were specifically bound to the bacteria, leading to the opening of the “gates” and the release of signal molecules (Figure 6B). A low detection limit of 17 cfu/mL was achieved.

A dual signal-on biosensor with the characteristics of accurate detection is also powerful in complex matrixes [79,80]. Coupling the benefits of the fluorescence technique with SERS, Wu et al. proposed a SERS and fluorescence dual-mode biosensor for AFB1 detection [81]. Briefly, R6G, acting as a fluorescence and Raman signal molecule, was embedded into the MSNs. Aptamers and polydopamine (PDA) were sequentially assembled on the surface of the MSNs as dual-gated molecules. In the presence of AFB1, the release of R6G was induced by the degradation of PDA and the specific binding between the aptamer and AFB1 (Figure 6C). Consequently, the detection limits for AFB1 detection by Raman and fluorescence spectroscopy were 0.133 pg/mL and 0.214 pg/mL, respectively. This biosensor exhibited good storage stability over a period of one week, indicating its good reusability and durability.

### 3.6. Mesoporous Silica-Based Chemiluminescence Biosensors

Chemiluminescence (CL) biosensors have also drawn much research attention due to their wide linear range, fast response, high sensitivity, simple operation, low background, and suitability for miniaturization [82,83,84]. Comparatively, CL relies on illumination through the chemical reactions of analytes with no need for a light source [85].

In a study by Sun et al. [86], a target-triggered CL sensor was proposed, based on a hollow MSN embedded with luminol by aptamers. In the presence of prostate-specific antigen, the prostate-specific antigen binds with its aptamer, leading to the release of luminol and triggering the chemiluminescence reaction of luminol-H_2_O_2_. Under optimized conditions, the sensor displayed a low detection limit of 0.27 pg/mL. The sensor also exhibited good selectivity, reproducibility, and stability, with a spiked recovery ranging from 99.3%–102.3% in serum samples without any further complex pretreatment.

**Figure 7:**
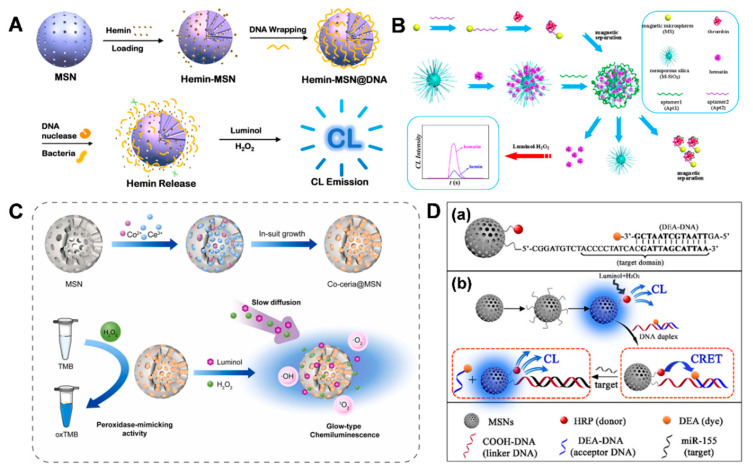
(**A**) Scheme of the stimulus-responsive CL platform for DNA nuclease and bacterial detection, adapted from Ref. [87] with the permission of the American Chemical Society. (**B**) Schematic CL sensor for thrombin detection, adapted from Ref. [88] with the permission of the American Chemical Society. (**C**) Schematic CL reaction of Co-ceria@MSN, adapted from Ref. [89] with the permission of Elsevier. (**D**) Scheme of CRET-based MSNs for miR-155 assay, adapted from Ref. [92] with the permission of the American Chemical Society.

To amplify the CL signal of a targeted analyte, Gu et al. prepared a CL biosensor for nuclease activity and bacterial determination using hemin-MSN@DNA [87]. Specifically, hemin was loaded into MSNs and then capped with the designed DNA. The capped DNA would be hydrolyzed under DNA nuclease, triggering the release of hemin to enhance the CL emissions for nuclease activity detection (Figure 7A). In addition, the nuclease derived from bacteria could also digest DNA to trigger CL enhancement.

To improve detection accuracy, a CL sensor with a dual-aptamer recognition effect was proposed for thrombin detection, based on MSNs that were encapsulated with iron porphyrin [88]. Briefly, MSNs were encapsulated with hematin by aptamer1 (Apt1/hematin/M-SiO_2_) and magnetic microspheres were modified with aptamer2 (Apt_2_/NH_2_-MS). In the presence of thrombin, Apt_2_/NH_2_-MS can recognize thrombin and separate it with a magnet, followed by further recognition of the separated thrombin-Apt_2_/NH_2_-MS by Apt1/hematin/M-SiO_2_, coupled with the simultaneous release of the encapsulated hematin to catalyze the CL reaction of luminol-H_2_O_2_ (Figure 7B). In this way, a sandwich-type CL sensor was constructed for thrombin detection.

To overcome the problems of detection accuracy and repeatability found in most flash-type CL biosensors, Qian et al. proposed a glow-type CL biosensor based on a bimetallic Co-doped ceria mesoporous nanocomposite (Co-ceria@MSN) [89]. This nanocomposite would act as a nanozyme to catalyze the luminol/H_2_O_2_ CL reaction, producing a CL signal (Figure 7C). Surprisingly, both the CL intensity and duration were strongly dependent on GSH concentration. When further applied for GSH determination, this CL platform presented a detection limit down to 10 nM.

In contrast to FRET, the CL resonance energy transfer (CRET) is generated from a chemical reaction without external excitation [90,91]. On the basis of using MSNs as carriers for embedding both the donor (HRP) and the acceptor (a functional DNA duplex), a new CRET biosensor for miRNA detection was designed by Shen et al. [92]. By controlling the energy-transfer distance, the donor emission could be quenched by the dye labeling the acceptor DNA. Upon the addition of the target miRNA, the CRET system was destroyed, followed by the release of the acceptor DNA due to the competitive hybridization of the target miRNA. Consequently, the CL signal was recovered (Figure 7D). This strategy offers a reference for biological assays.

## 4. Summary and Outlook

In this review, we discussed the latest developments in MSN-based biosensing strategies from the preparation of MSNs to biosensing applications, including colorimetry, fluorescence, electrochemistry, ECL, SERS, and chemiluminescence biosensors.

Despite extensive research on MSNs, most of the studies on MSNs are still in their infancy. The prospects for their practical applications are infinite, and further research efforts need to focus on the following issues.

(I) In terms of preparation methods, more controllable strategies should be explored with a desirable structure/composition.
(II) In terms of diagnostics, MSNs act as nanocarriers without orientation, so research efforts should be made to improve their ability to combine with other substances and enhance detection accuracy [93]. Meanwhile, their specificity cannot be ignored in the performance evaluation of biosensors.
(III) In terms of safety, although the toxicity of MSNs is probably low, their long-term toxicity is still unknown. Sufficient attention should be paid to decreasing their toxicity and accelerating their degradability in biological systems.
(IV) In terms of application research, in-depth studies on other biosensing applications of MSN nanomaterials are urgently needed. There is still room for the development of biosensing applications based on MSNs, and these may provide references for related researchers.
(V) Currently, most detection systems are still in their infancy, and their practical application remains a challenge. Future studies should focus on their practical application, especially the development of portable instruments [94].
(VI) MSNs have garnered much attention due to their merits, including high stability and ease of loading, which make them possible for clinical translation. The preparation of highly homogeneous MSNs with a low molecular weight is a prerequisite for promoting their clinical application.
(VII) The multifaceted capabilities of MSNs provide possibilities in terms of simultaneously detecting multiple targets. Thus, the design of sensing arrays, combined with microfluidic technology, may expand the frontiers of their applicability. Furthermore, integrating colorimetry with smartphones is promising for improving the portability of biosensing.
